# Very low birth weight infants in China: the predictive value of the motor repertoire at 3 to 5 months for the motor performance at 12 months

**DOI:** 10.1016/j.earlhumdev.2016.03.010

**Published:** 2016-09

**Authors:** Fei-Fei Zang, Hong Yang, Qian Han, Jia-Yan Cao, Iris Tomantschger, Magdalena Krieber, Wei Shi, Dan-Dan Luo, Mo Zhu, Christa Einspieler

**Affiliations:** a Department of Rehabilitation, Children's Hospital of Fudan University, Key Laboratory of Neonatal Diseases, Ministry of Health, Shanghai, PR China; b Health Service Center, Meilong Community, Minhang District, Shanghai, PR China; c Research Unit iDN – interdisciplinary Developmental Neuroscience, Institute of Physiology, Center for Physiological Medicine, Medical University of Graz, Austria

**Keywords:** General movements, Motor optimality score, Very low birth weight infants, Preterm infant, Reliability

## Abstract

**Background:**

Studies on motor performance and its early markers are rare in China, especially in very low birth weight (VLBW) infants.

**Objective:**

Apart from the assessment of the inter-scorer agreement, we aimed to analyze to what extent the motor repertoire at 10 to 18 weeks postterm was related to neonatal complications, and gross and fine motor performance at 12 months after term.

**Study design:**

Exploratory prospective study.

**Subjects:**

Seventy-four VLBW infants (58 males; mean gestational age = 29 weeks; mean birth weight = 1252 g).

**Method:**

Five-minute video recordings were performed at 10 to 18 weeks after term; fidgety movements and the concurrent motor patterns (resulting in a motor optimality score) were assessed according to the Prechtl general movements assessment (GMA). The gross and fine motor performance was assessed by means of the Peabody Developmental Motor Scales, second edition, at 12 months.

**Results:**

Reliability was excellent. Pneumonia was associated with absent fidgety movements; the motor optimality score was lower in infants with pneumonia and/or bronchopulmonary dysplasia. Both absent fidgety movements and a lower motor optimality score were associated with a poor or very poor gross and fine motor performance at the 12-month-assessment.

**Conclusion:**

Both the assessment of fidgety movements and the evaluation of the concurrent motor repertoire contribute significantly to an identification of VLBW children with a poor gross and fine motor outcome at 12 months. The results of this study document the need for an early identification of infants at high risk for a poor motor performance.

## Highlights

• Postures and movements at 10 to 18 weeks after term could be reliably assessed in very low birth weight infants in China.
• If fidgety movements did not develop, gross and fine motor performance was poor at age 1.
• A detailed assessment of motor behaviour adds to the assessment of fidgety movements.

## 1 Introduction

Research on the development of very low birth weight (VLBW) infants has come to the fore in recent years, but there still appears to be little or no data available from China [1], [2], [3] albeit there are more than 100,000 infants born with VLBW annually [4]. Although the survival rate of VLBW infants has been greatly improved, they are still more likely to develop neurological deficits. Studies on large samples show that 25% to 50% of VLBW infants will develop cognitive and/or behavioral deficits, and 5% will develop cerebral palsy or other neuromotor impairments [3], [5], [6]. Hence, an early identification of infants at increased risk for neurological deficits is of utmost importance. The Prechtl general movements assessment (GMA) is a non-invasive, reliable and valid method for an effective assessment of the function of the young nervous system [7], [8], [9], [10]. General movements (GMs) are spontaneous movements that emerge at 9 weeks postmenstrual age and last until 5 months after term. They vary in intensity and speed, and involve the entire body with variable sequences of neck, trunk, arm, and leg movements. Preterm GMs are followed by writhing GMs around term age. By the end of the second month after term, writhing movements gradually disappear and are then replaced by fidgety GMs, which are tiny movements of the neck, trunk, and limbs in all directions, with small amplitude, moderate speed, and variable acceleration [7], [8], [11].

Apart from classifying GMs as “normal” or “abnormal” (i.e. the global GMA), GMs and the concurrent motor repertoire can be assessed in more detail [7], [12]. The detailed scoring of the motor repertoire at 3 to 5 months focuses on the quantity and quality of various movements including fidgety GMs, on postural patterns, and the overall movement character [7], [13], [14]. It has a high inter-rater reliability with intra-class correlation coefficients (ICC) ranging from 0.80 to 0.94 [15]. The scoring list has been successfully used to predict the severity of cerebral palsy [16], [17], or to identify an increased risk for minor neurological dysfunction [13], [18] and suboptimal cognitive development [19], [20] in infants born preterm. It has also been used to demonstrate that, e.g., prenatal exposure to polychlorinated biphenyls [21] or selective serotonin reuptake inhibitors [22] have an impact on the developing nervous system.

The aim of our study was to assess Chinese infants with VLBW at 10 to 18 weeks after term by means of both global and detailed GMA and to associate these findings with their motor performance around 12 months (corrected for preterm birth). Apart from (a) the assessment of the inter-scorer agreement, we specifically aimed to (b) analyze to what extent the motor repertoire at 10 to 18 weeks was related to (i) neonatal complications, and (ii) the gross and fine motor performance at 12 months postterm age.

## 2 Methods

### 2.1 Participants

A successive sampling method was used to recruit preterm infants who met the following criteria: (i) birth weight under 1500 g; (ii) born between September 1, 2011 and August 31, 2013; (iii) seen for a visit at the Department of Rehabilitation of the Children's Hospital of Fudan University, Shanghai, PR China, at an age of 10 to 18 weeks after term. The following exclusion criteria applied: infants with brain malformations, a chromosomal defect, or known syndrome. A total of 77 infants were found to be eligible for the study. Three infants were excluded because their neonatal core data were incomplete. The final sample comprised 74 children (58 male, 16 female). The gestational age at birth ranged from 24 to 34 weeks (mean = 29 weeks, SD = 2 weeks); their mean birth weight was 1252 g (SD = 210 g; range: 700 to 1495 g). The neonatal complications included brain injury (44.6%; in 21 infants [28.4%] the clinical protocol only lists “abnormal brain image” without further specification; 12 infants [16.2%] had intraventricular haemorrhage grade III/IV or white matter abnormalities), respiratory distress syndrome (32.4%), septicaemia (14.9%), bronchopulmonary dysplasia (BPD, 10.8%), pneumonia (9.5%), and necrotizing enterocolitis (6.8%); most of the infants had multiple complications.

### 2.2 Assessment of the motor repertoire at 10 to 18 weeks postterm age

The infants were videoed at a median age of 13 weeks (range: 10 to 18 weeks) postterm. Recordings were made during active wakefulness according to the standards of the Prechtl GMA [7], [23]. Periods of fussing, crying, hiccupping, and sucking on a dummy were excluded. The average duration of the video recordings available for analysis was 5 min (range: 2 to 10 min).

Fidgety movements were scored as (a) present and normal; (b) present and abnormal; or (c) absent [7], [8], [11]. Infants who showed “sporadic fidgety movements” (i.e. isolated bursts of fidgety activity lasting < 3 s) were classified as “absent fidgety movements” [24]. The detailed scoring consists of the assessment of movement patterns (24 items), postural patterns (13 items), and the observation of the movement character (10 items). The score sheet comprises the following five subcategories: (i) fidgety movements, (ii) age-adequacy of motor repertoire, (iii) quality of movement patterns other than fidgety movements, (iv) posture, and (v) overall quality of the motor repertoire [7], [13], [14]. Added up, the subcategories yield a total score of 28 to 5 (the “motor optimality score”), the maximum score indicating the best performance [7], [13], [25].

This score sheet was translated from English into Mandarin by a rehabilitation doctor (M.Z.); it was proofread and modified, where necessary, by a senior paediatrician (H.Y.), who is a licensed tutor for GMA. Finally, the Mandarin version was re-translated into English and reviewed by another licensed tutor for GMA (C.E.), who also provided training for the detailed assessment.

To assess inter-scorer agreement, three scorers, all of whom were trained and certified in the Prechtl GMA (basic training level), performed the detailed GMA of 30 infants (40.5%). Scorer 1 (F.F.Z.) is a PhD student with her focus on GMA; prior to launching the study, she assessed GMs for more than a year with an average of 40 infants per week. Scorer 2 (Q.H.) is a paediatrician working in a community department and had used GMA for 2 years, assessing approximately 15 infants per week. Scorer 3 (J.Y.C.) is a physiotherapist at the Children's Hospital of Fudan University; she had applied the GMA for more than a year on an average of 40 infants per week. All three scorers had been trained in the detailed assessment by C.E. All scorers independently assessed the 30 videos without any knowledge of the medical history of the infants. During assessment, they were allowed to watch the videos as often as necessary.

After finishing the individual assessments, Scorers 1 to 3 discussed disagreements with a licensed tutor for GMs (H.Y., who was also unfamiliar with the infants' medical history) and agreed on a final motor optimality score for each infant. The remaining 44 infants were assessed by all scorers together.

### 2.3 The outcome assessment

At a median postterm age of 12 months (range: 12 to 18 months), we applied the Peabody Developmental Motor Scales, second edition (PDMS-2; [26]; translation into Mandarin [27]) in order to assess qualitative and quantitative aspects of gross and fine motor development. The scales contain sub-tests of the following six parameters: (a) reflexes, (b) stationary (body control and equilibrium), (c) locomotion, (d) object manipulation, (e) grasping, and (f) visual–motor integration. Raw scores are converted into age-equivalent scores for each sub-test, percentiles, sub-test standard scores, and composite standard scores called motor quotients. For children older than 1 year, the results from (b), (c) and (d) generate the Gross Motor Quotient (GMQ); the results of (e) and (f) yield the Fine Motor Quotient (FMQ); the sum of GMQ and FMQ reveals the Total Motor Quotient (TMQ). Although the PDMS-2 has a mean motor quotient standard score of 100 and a standard deviation (SD) of 15, it classifies performance primarily based on 10-point increments (rather than the 15-point SD increments) into the following categories: very superior (standard score: 131–165), superior (standard score: 121–130), above-average (standard score: 111–120), average (standard score: 90–110), below average (standard score: 80–89), poor (standard score: 70–79), and very poor (standard score: 35–69) [26].

The assessment was performed by two paediatricians (D.D.L. and W.S.)—without knowledge of the medical history and the results of the GMA—at the Department of Rehabilitation, Children's Hospital of Fudan University.

### 2.4 Statistics

Statistical analysis was performed using the SPSS package for Windows, version 22.0 (SPSS Inc, Chicago, IL). Intra-class correlation coefficient (ICC) statistics were applied to examine pairwise agreement of the motor optimality scores among the three scorers and an overall agreement among all scorers.

Fisher's exact test was applied to compare nominal data (e.g. neonatal complication × fidgety movements). The independent samples *T*-test was used to compare whether two groups (e.g. with absent or present fidgety movements) have different average values of birth weight and gestational age. The Mann–Whitney *U* test was applied to compare two groups with regard to neonatal complications on one dependent outcome variable (i.e. motor optimality score). Linear-by-linear association was applied to assess the relation between nominal variables (e.g. fidgety movements) and ordinal variables (i.e. categories of PDMS-2). Spearman's rank order correlation was applied to correlate ordinal variables (i.e. categories of PDMS-2) with metric scales (e.g. motor optimality score). To assess the relation between two metric variables (e.g. birth weight and motor optimality score), we applied Pearson's product–moment correlation coefficient.

### 2.5 Ethics

The study was approved by the Medical Ethics Committee of the Children's Hospital of Fudan University, Shanghai, PR China. The infants' parents gave their written informed consent to their infants' participation in the study.

## 3 Results

### 3.1 The assessment of the motor repertoire at 10 to 18 weeks postterm age and its reliability

Fifty-seven infants (77%) had normal fidgety movements, while 17 infants had no fidgety movements (23%); none of the participants showed abnormal fidgety movements. All scorers agreed on the presence/absence of fidgety movements (100% inter-scorer agreement).

The median motor optimality score was 24 (P25 = 22, P75 = 26; range: 7 to 28). Reliability was excellent (all ICCs > 0.90; Table 1).

### 3.2 Neonatal complications and their association with the motor repertoire at 10 to 18 weeks postterm age

Infants aged 10 to 18 weeks with no fidgety movements had tended to have lower birth weight than infants who developed fidgety movements (*p* < 0.10; Table 2). Of all listed neonatal complications, only pneumonia (mainly ventilator-associated) correlated with absent fidgety movements (*p* < 0.01; Table 2). Accordingly, the motor optimality score was lower in infants with pneumonia (*p* < 0.01) and/or BPD (*p* < 0.05; Table 3). The motor optimality score was significantly related to birth weight (*r* = 0.234, *p* < 0.05), but not to gestational age (*r* = 0.160, *p* = 0.17).

### 3.3 Association between the motor repertoire at 10 to 18 weeks postterm and the 1-year outcome

Due to the long distance between their hometown and Shanghai, six families withdrew from participation at the follow-up assessment. Hence, sixty-eight 12- to 18-month-olds were tested by means of PDMS-2. The latter did not differ from the drop-outs according to fidgety movements (*p* = 0.13) or their motor optimality score (*p* = 0.82).

**Fig. 1:**
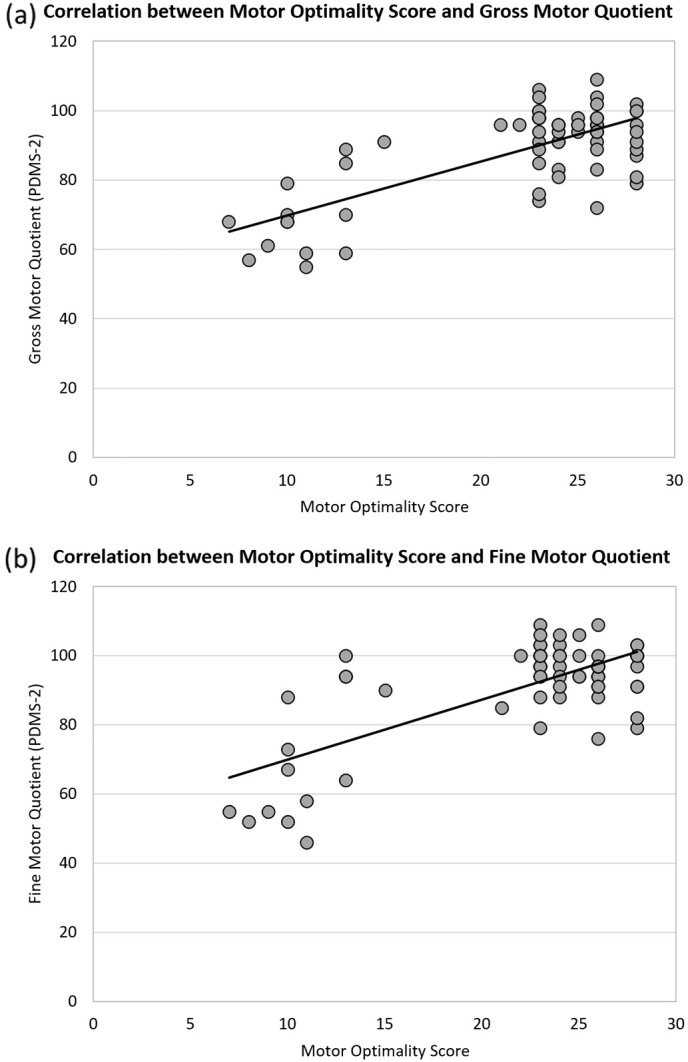
Correlation between the motor optimality score obtained at 10 to 18 weeks after term and the Peabody Developmental Motor Scales' (second edition; PDMS-2) scores at 12–18 months (*n* = 68).

The median GMQ of the PDMS-2 was 94 (P25 = 81, P75 = 98; range: 55 to 109): the median FMQ was 96 (P25 = 88, P75 = 100; range: 46 to 109) revealing a median TMQ of 93 (P25 = 80, P75 = 97; range: 47 to 108). Individuals with normal fidgety movements scored higher than individuals without fidgety movements with respect to TMQ, GMQ, and FMQ (Table 4). Fig. 1 shows that a low motor optimality score was associated with a poor performance in the PDMS-2 (GMQ: rho = 0.413; FMQ: rho = 0.326; TMQ: rho = 0.406; all *p*s < 0.01).

According to the TMQ, 46 individuals (67.6%) scored average, five (7.4%) scored below average, ten (14.7%) scored poor, and seven (10.3%) scored very poor (Table 5). Out of those 22 individuals with a TMQ below average, twelve had no fidgety movements while ten had normal fidgety movements. Four of the latter missed the optimality criterion (i.e. a motor optimality score of at least 26 points; [25]; Table 5). Hence, we identified them as moving less optimal during infancy although their fidgety movements were normal.

As regards gross motor performance, 46 individuals (67.6%) scored average, seven (10.3%) scored below average, seven (10.3%) scored poor, and eight (11.8%) scored very poor.

With respect to fine motor performance, 50 individuals (73.5%) scored average, six (8.8%) scored below average, four (5.9%) scored poor, and eight (11.8%) scored very poor.

Adding the motor optimality score to the assessment of fidgety movements enabled the identification of five or four individuals at risk for below-average gross or fine motor performance at 12 to 18 months, respectively (Table 5).

## 4 Discussion

Our study on VLBW Chinese infants confirms that the absence of fidgety movements is associated with a poor gross and fine motor performance as assessed by the PDMS-2. It was the first study to apply the detailed GMA in a sample of Chinese high-risk infants.

As the GMA is based on visual Gestalt perception, a high inter-observer agreement is vital. In their review, Einspieler and Prechtl [8] reported agreement rates of 89% to 93% among 90 observers, and an average Cohen kappa of 0.88. In 2007, Yang et al. [28] conducted a reliability study on the global GMA in 58 high-risk Chinese infants and obtained ICCs of 0.97 to 0.99, and a re-test ICC of 0.69. Whereas agreement between experienced scorers was excellent, beginners were only moderately reliable [28]; similar results were reported in Switzerland [29]. There has only been one reliability study on detailed GMA to reveal ICCs > 0.87, which indicates that the assessment of the four scorers was reliable [15]. Our results testify that detailed GMA is a reliable tool for trained observers (ICCs > 0.90).

Previous studies carried out in Germany [30], South Africa [31] and Australia [32] demonstrate that the assessment of fidgety movements contributes to the predictability of the motor outcome in VLBW or very preterm infants. Like in Spittle et al. [32] a significant percentage of infants in our study developed no fidgety movements (22% of 97 Australian very preterm infants; 23% in our study). In the South African sample of 125 VLBW infants, only 9% had no fidgety movements [31]. Similarly, in the sample of Stahlmann et al. [30] only 8% of 103 VLBW infants showed no fidgety movements. However, there is a significant discrepancy between the clinical data of the studies, with 45% of infants showing brain injury in our study, as opposed to 11% in the Australian study [32]; also, the rate of BPD (11%) or septicaemia (15%) was higher in our sample than, for example, in the German sample (BPD in 6%, septicaemia in 11%) [30]. On the other hand, the rate of neonatal complications in our sample was similar to the one reported for the Chinese population [3], [33], [34]. Whereas brain injury was not associated with the motor optimality score at 10 to 18 weeks, BPD and (ventilation-associated) pneumonia were. Hitzert et al. [35] found a considerable number of preterm infants with BPD who developed no fidgety movements, though this was mainly due to dexamethasone treatment and hardly concerned infants treated with hydrocortisone [35], [36]. Unfortunately, our data do not allow for such a comparison as we have no access to the details of postnatal corticosteroid therapy.

At the 1-year assessment, none of the participants of our sample scored above average. The percentage of PDMS-2 scores “below average” was similar to the study by Burger et al. [31], though with a higher rate of “poor” or “very poor” scores. Lee et al. [37] demonstrated that Asian children with VLBW generally have lower PDMS-2 scores than the normative sample from the USA and Canada, though it must be noted that Lee et al. applied the PDMS-2 at 3 and 5 years of age.

Table 4 demonstrates a significant association between the presence/absence of fidgety movements and the PDMS-2 scores, although we also found five infants (9.3%) with fidgety movements who scored poorly in the 1-year PDMS-2 assessment (Table 5). By incorporating the optimality criterion, the proportion of individuals who remained undetected could be further reduced to 5.6%. Hence, the assessment of the additional motor repertoire (postures and movements other than fidgety movements) adds to the assessment of fidgety movements. Apart from an early identification of infants at high risk for motor dysfunctions, a careful and detailed evaluation of movement and postural patterns enables us to find strengths and limitations in each child that might induce individual intervention strategies.

### 4.1 Limitations

Carrying out an outcome assessment at the age of 1 isn't ideal, but then several authors have associated the results of GMA with the motor outcome of preterm-born children at 1 year [31], [38], [39]. A shortcoming of our study is that no neurological examination was performed at the 12-month appointment. Nor is the information about brain injury standardised; in a number of cases, the clinical protocol only lists “abnormal brain image” without specification. On the other hand, GMA contributes to the neurological examination and helps to predict the motor outcome when brain imaging is not available. This is a benefit for less privileged countries, as was previously shown in South Africa, Iran, or Brazil [31], [39], [40].

## 5 Conclusion

In addition to the high inter-scorer agreement for the Chinese translation of the score sheet for the detailed GMA, we were able to confirm that the assessment of fidgety movements and the concurrent motor repertoire at 10 to 18 weeks postterm helps to identify VLBW infants at an increased risk for poor or very poor gross and fine motor performance at 12 (to 18) months. Early identification enables early intervention programmes before pathological features become manifest. Follow-up programmes for preterm infants are still rare in China. The results of the present study document that there is a need in China and other low- and middle-income countries for early identification of infants at risk for poor motor performance. On the other hand, a normal early motor behavior will also give arguments to physicians to reassure parents not to start intervention but provide the scarce means to those who need it most.

## Figures and Tables

**Table 1:** Intra-class correlation coefficients (ICC; 95% confidence interval) providing inter-scorer agreement on the motor optimality scores of 30 infants, pair-wise between three scorers and for all scorers.

	ICC (95% confidence interval)	*p*-value
Scorer 1–Scorer 2	ICC = 0.970 (0.935–0.986)	*p* < 0.01
Scorer 1–Scorer 3	ICC = 0.986 (0.971–0.993)	*p* < 0.01
Scorer 2–Scorer 3	ICC = 0.974 (0.947–0.988)	*p* < 0.01
All scorers	ICC = 0.977 (0.958–0.988)	*p* < 0.01

**Table 2:** Neonatal characteristics and complications, and their association with fidgety movements at 10 to 18 weeks.

Neonatal characteristics and complications	Fidgety movements		*p*-value^a^
Present (*n* = 57)	Absent (*n* = 17)
Male	43 (75.4%)	15 (88.2%)	0.33^a^
Gestational age	Mean = 29.5 wks	Mean = 29 wks	0.45^b^
(SD = 2 wks)	(SD = 2.5 wks)
Birth weight	Mean = 1275 g	Mean = 1175 g	0.09^b^
(SD = 200 g)	(SD = 232 g)
Brain injury^c^	23 (40.3%)	10 (58.8%)	0.27^a^
Respiratory distress syndrome	18 (31.5%)	6 (35.2%)	0.78^a^
Septicaemia	8 (14.0%)	3 (17.6%)	0.71^a^
Bronchopulmonary dysplasia	5 (8.7%)	3 (17.6%)	0.37^a^
Pneumonia	2 (3.5%)	5 (29.4%)	< 0.01^a^
Necrotizing enterocolitis	2 (3.5%)	3 (17.6%)	0.09^a^

^a^Fisher's exact test; ^b^Independent samples *T*-test; ^c^According to cranial ultrasound and MRI; MRI was performed in 49 individuals (66.2%); in 11 individuals, abnormal MRI was documented, but no further specification was given.

Key: SD = standard deviation; wks = weeks

**Table 3:** Neonatal complications and their association with the motor optimality score at 10 to 18 weeks.

Neonatal complications	Motor optimality score median (P25–P75)		*p*-value
Present	Absent
Brain injury^b^	23	24	0.36^a^
(13–26)	(23–26)
*n* = 33	*n* = 41
Respiratory distress syndrome	24	24	0.84^a^
(17–26)	(22–26)
*n* = 24	*n* = 50
Septicaemia	24	24	0.72^a^
(15–28)	(22–26)
*n* = 11	*n* = 63
Bronchopulmonary dysplasia	22	24	< 0.05^a^
(15–23)	(23–26)
*n* = 8	*n* = 66
Pneumonia	11	24	< 0.01^a^
(10–23)	(23–26)
*n* = 7	*n* = 67
Necrotizing enterocolitis	10	24	0.25^a^
(8–27)	(23–26)
*n* = 5	*n* = 69

^a^Mann–Whitney *U* test; ^b^See legend to Table 1.

Key: P = percentile rank.

**Table 4:** Association between fidgety movements and the Peabody Developmental Motor Scales (second edition; PDMS-2); motor quotients are given in median (P25–P75) and range.

PDMS-2	Fidgety movements		*p*-value
Present	Absent
(*n* = 54)⁎	(*n* = 14)⁎
Gross motor quotient	Median = 96	Median = 68	*p* < 0.01^a^
(91–98)	(59–81)
72–109	55–91
Fine motor quotient	Median = 97	Median = 66	*p* < 0.01^a^
(93–100)	(54–91)
76–109	46–100
Total motor quotient	Median = 94	Median = 68	*p* < 0.01^a^
(92–98)	(55–76)
71–108	47–93

Key: PDMS-2 = Peabody Developmental Motor Scales, second edition.

⁎ six individuals did not participate in the PDMS-2; hence the sample size is only 68.

a Mann–Whitney *U* test.

**Table 5:** Peabody Developmental Motor Scales (second edition; PDMS-2) classifications depending on fidgety movements and motor optimality score.

PDMS-2	Fidgety movements × Motor optimality score			
Absent FMs, MOS < 26 (*n* = 14)*	Present FMs, MOS < 26 (*n* = 29)*	Present FMs, MOS 26–28 (*n* = 25)*	*p*-value
Total motor performance				rho = 0.455^a^ *p* < 0.01
Average	2 (14.3%)	25 (86.2%)	19 (76%)
Below average	0	2 (6.9%)	3 (12%)
Poor	5 (35.7%)	2 (6.9%)	3 (12%)
Very poor	7 (50%)	0	0
Gross motor performance				rho = 0.556^a^ *p* < 0.01
Average	1 (7.1%)	24 (82.8%)	21 (84%)
Below average	2 (14.3%)	3 (10.3%)	2 (8%)
Poor	3 (21.4%)	2 (6.9%)	2 (8%)
Very poor	8 (57.1%)	0	0
Fine motor performance				rho = 0.429^a^ *p* < 0.01
Average	4 (28.6%)	25 (86.2%)	21 (84%)
Below average	1 (7.1%)	3 (10.3%)	2 (8%)
Poor	1 (7.1%)	1 (3.4%)	2 (8%)
Very poor	8 (57.1%)	0	0

Key: FMs = fidgety movements; MOS = motor optimality score; PDMS-2 = Peabody Developmental Motor Scales, second edition.

a Spearman's rank order correlation coefficient.
